# Pharmacist Reported Protocols for QTc Monitoring of Psychiatric Medications

**DOI:** 10.7759/cureus.57192

**Published:** 2024-03-29

**Authors:** Kathleen Harb, Shaina Schwartz, Julie Cooper

**Affiliations:** 1 Clinical Sciences, High Point University Fred Wilson School of Pharmacy, High Point, USA

**Keywords:** psychiatric medication, cardiology, monitoring, pharmacist, qtc interval

## Abstract

Background

Psychiatric medications, such as antipsychotics and antidepressants, are associated with QTc interval prolongation. There is currently no consensus best practice on how to mitigate this risk. This study aimed to collect and analyze information about methods used for QTc monitoring in patients taking psychiatric medications to better understand current practice.

Methods

An anonymous electronic survey was distributed on September 22, 2022, using a national psychiatric pharmacist organization email list. The survey closed on December 15, 2022. Descriptive statistics were used to analyze the multiple-choice questions. Qualitative analysis applying grounded theory for thematic analysis was performed for free response questions.

Results

A total of 48 initiated the survey. Of the respondents, 11.4% (5/44) reported that their institution had a formal protocol for monitoring QTc intervals in patients receiving psychiatric medications, while 32.4% (12/37) reported that their institution had an informal process. Out of those with a protocol or process, approximately half reported that it was drug-specific. Among the respondents, 88.6% (31/35) reported that there was a psychiatric clinical pharmacy specialist at their institution and 34.3% (12/35) reported that pharmacists could order an electrocardiogram (ECG). Major themes that emerged from the qualitative analysis included pharmacist-driven QTc monitoring, referring the patient to another provider for monitoring, and encountering significant barriers to monitoring.

Conclusion

A variety of methods are currently being employed to monitor QTc prolongation risk in patients taking psychiatric medications. Pharmacist authorization to order ECGs may be an opportunity to advance practice and improve care for this population. Further research is needed to more clearly understand best practices for QTc prolongation risk mitigation in patients receiving psychiatric medications.

## Introduction

Many psychiatric medications have an associated risk of prolonged QTc interval which may lead to torsades de pointes (TdP), a serious cardiac arrhythmia that can cause sudden death. These medications belong to various therapeutic classes including antipsychotics and antidepressants [1]. In addition to the medications, there are many patient-specific risk factors such as age, genetics, female sex, cardiovascular history, renal or hepatic dysfunction, and electrolyte imbalances that must be considered when determining a patient's risk for QTc prolongation and TdP [2,3]. For every 10 milliseconds (msec) above a QTc of 400 msec, the risk of TdP increases proportionally [4]. A QTc interval of >460 msec in women or >450 msec in men is considered to be prolonged and a QTc interval of >500 is considered to be high risk in either sex [5]. An example of how medications can impact the QTc interval can be seen in a 2016 study which showed that the rate of QTc prolongation was 71% in men taking QTc prolonging medications versus 48% for men who were not, compared with 50% versus 34% for women, respectively [6]. A study on the prescribing prevalence of QTc prolonging medications found that, out of 1.1 million patients that filled prescriptions for QTc prolonging medications, 9.4% filled prescriptions for 2 or more of those medications or for a QTc interval-prolonging agent and a drug that decreases its clearance [7]. Concurrent use of multiple QTc prolonging medications may further increase a patient's risk for QTc prolongation and TdP [4]. Polypharmacy of antidepressants and antipsychotics has been increasing which raises further concern about this issue [8,9].

In addition to taking medications with a known QTc interval prolonging risk, the psychiatric patient population also has an increased cardiovascular risk compared to the general population [10]. This myriad of risk factors makes the need for QTc interval assessment guidelines paramount in the psychiatric patient population. A recent study found that out of 169 patients prescribed antipsychotics, 52.1% had at least one abnormal ECG, and 20.7% had two or more abnormal ECGs [11]. Another study showed that the prevalence of ECG abnormalities in hospitalized psychiatric patients was 17.9%, including 7.6% with a prolonged QTc [12]. In addition to patient-specific risk factors, there are also health system barriers to QTc interval monitoring and management in this population. One literature review found a few of these barriers to be a lack of knowledge and expertise among providers on when or how to monitor, a lack of available resources, and fragmentation of care between general practitioners and psychiatrists [13]. These issues make the need for clear guidelines surrounding QTc monitoring in psychiatric patients even more important. 

Guidelines refer to a set of information that is used to inform a practitioners’ decision making while a protocol is a clear set of rules that may include a step-by-step algorithm. The application of guidelines can vary between individual providers, practice settings, or institutions. Protocols do not allow for as much deviation as they prescribe what action is needed based on a set of circumstances. An example of a clear algorithm used in most institutions is the advanced cardiac life support (ACLS) algorithm for basic life support [14]. There are guidelines available for when and how to assess the QTc interval to avoid TdP from the American Heart Association (AHA) and the American Psychiatric Association (APA), but it is unclear if either is commonly used in practice in the United States [15,16]. The AHA guidelines focus on hospitalized patients, and include information on patient-specific risk factors and a few medications that are known to cause QTc prolongation. The APA guidelines go into depth on many of the psychiatric medications and medication classes that are known to cause QTc prolongation while also stressing the importance of patient-specific considerations. There are also validated clinical scoring tools to assist providers in determining risk such as the Tisdale score [17]. Some hospitals have developed their own institution-specific systems such as the computer-based ECG screening system developed and implemented at Mayo Clinic [18]. 

Currently, there is no consensus on best practices on how to mitigate the risk of QTc prolongation associated with the use of psychiatric medications. There is controversy surrounding whether and when the QTc interval should be routinely monitored due to financial and logistical barriers, especially considering that TdP is an uncommon occurrence [19,20]. Practitioners' fear of TdP can lead to frequent monitoring that can be expensive and time-consuming and may be distressing to the patients [20]. While some monitoring may be unnecessary, there are clear situations where QTc monitoring is needed. Due to the lack of a widely accepted best practice or guideline, hospitals across the country could vary significantly in their monitoring recommendations. To our knowledge, no previous study has been conducted to evaluate hospital QTc monitoring protocols for patients prescribed psychiatric medications. This study aimed to collect and analyze protocols for QTc monitoring in patients taking psychiatric medications to better understand current practice strategies and their concordance with existing guidelines.

This research was previously presented as a meeting abstract and poster at the 2023 American Association of Psychiatric Pharmacists (AAPP) Annual Meeting on April 17, 2023.

## Materials and methods

An electronic survey assessing QTc monitoring protocols was developed using Qualtrics software (Qualtrics, Provo, UT, USA). The survey included nine questions that consisted of a mixture of multiple-choice and free-response questions. Multiple-choice questions had the following response options: Yes, No, Not Sure, and Other (which included a text box to appear so that respondents could elaborate). The survey included logic so that if the respondents answered "yes" to the questions about having a formal or informal protocol or process for monitoring, they would then be prompted to briefly describe the protocol or process and/or email a copy to one of the investigators. Questions asking for specific details ("Is your protocol/process drug-specific?" and "Does your protocol/process utilize a risk assessment method and/or scoring tool (e.g. Tisdale)?") were only displayed if the respondent indicated that their institution possessed a process or protocol. The content of questions centered around the existence of a protocol, the details of the protocol, and the pharmacist's ability to order ECGs. Questions developed and chosen to be included in the survey were ones the authors determined to be the most clear and concise to avoid confusion or misinterpretation. See Table 1 for the full list of multiple-choice questions used.

**Table 1:** Quantitative analysis of survey results ECG = electrocardiogram

Question	Number of Yes responses (%)	Number of No responses (%)	Number of Not Sure responses (%)	Number of Other responses (%)	Total number of respondents
Does your institution have a formal protocol for monitoring QTc interval in patients prescribed psychiatric medications?	5 (11.36)	36 (81.82)	1 (2.27)	2 (4.55)	44
Does your institution have an informal process for monitoring patients' prescribed medications with a risk of causing QTc prolongation?	12 (32.43)	20 (54.05)	3 (8.11)	2 (5.41)	37
Is your protocol/process drug-specific?	5 (45.45)	6 (54.55)	0 (0)	0 (0)	11
Does your protocol/process utilize a risk assessment method and/or scoring tool (e.g. Tisdale)?	4 (36.36)	6 (54.55)	1 (9.09)	0 (0)	11
Can pharmacists order an ECG at your institution?	12 (34.29)	22 (62.86)	1 (2.86)	0 (0)	35
Do you have a psychiatric clinical pharmacy specialist at your institution?	31 (88.57)	3 (8.57)	0 (0)	1 (2.86)	35

Institutional review board (IRB) approval for this study was obtained on January 18, 2022, from the affiliated university. The IRB approved this project under exempt review category 4, secondary research for which consent is not required. A link to the survey was distributed on September 22, 2022, using a national psychiatric pharmacist organization email list to 740 subscribers. The survey remained open until December 15, 2022. Participation was entirely voluntary. All submitted survey responses were anonymous and all responses were included in the analysis. Microsoft Excel (Microsoft Corporation, Redmond, WA, USA) was used to export survey data for statistical analysis. Descriptive statistics were used to analyze multiple-choice questions. Qualitative analysis applying grounded theory for thematic analysis was performed for the free-response questions. Free-response questions were independently coded for themes by two investigators. The themes were reviewed by the coding investigators to agree on thematic codes and resolve initial discrepancies. A third investigator reviewed the thematic coding of the data using the final codebook for validation.

## Results

A total of 48 individuals initiated the survey, yielding a 6.5% (48/740) response rate. A survey was considered to be initiated if at least one question was answered before submission. Responses per question varied from 44 respondents to 11 respondents, with the question about whether or not the institution had a formal protocol for QTc monitoring having the highest response rate and the questions about having a drug-specific protocol or using a risk assessment method having the lowest response rate. Of the respondents, 11.4% (5/44) reported that their institution had a formal protocol for monitoring QTc intervals in patients receiving psychiatric medications, while 32.4% (12/37) reported that their institution had an informal protocol. Out of those with a formal or informal protocol, approximately half reported it was drug-specific and did not include a risk assessment or scoring tool. Of the respondents, 88.6% (31/35) reported there was a psychiatric clinical pharmacy specialist at their institution, and 34.3% (12/35) reported that pharmacists could order an ECG. The variation in response rates for each question may affect the reliability and interpretation of the data. The variation may be indicative of areas of uncertainty or poorly written questions.

**Figure 1:**
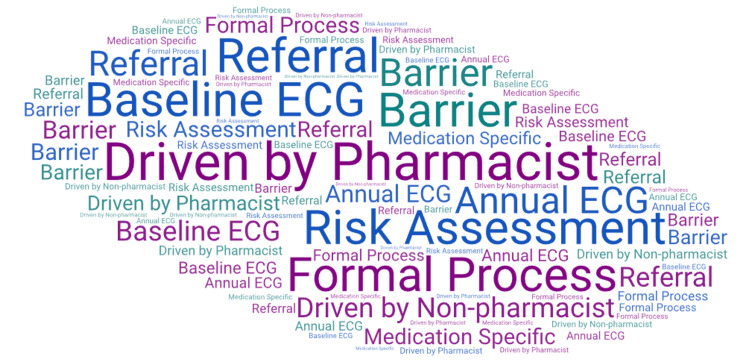
Word cloud of survey responses

If a participant answered "other" to one of the questions, they were given the option to provide more information in a free text portion of the survey. For the question about having a formal protocol, one participant wrote, "Not institution wide yet. At the sister hospital, pharmacists monitor all high risk meds for QTc prolongation". For the question about having an informal process, one participant wrote, "If pharmacist or provider recommends ECG". For the question about having a psychiatric clinical pharmacy specialist at the institution, one participant wrote, "Only a non-employed pharmacy faculty member". Major themes that emerged from the qualitative analysis included pharmacist-driven QTc monitoring, referring the patient to another provider for monitoring, and encountering significant barriers to monitoring. See Figure 1 and Table 2 for data from the qualitative analysis. Figure 1 describes the qualitative data in a word cloud format. Each theme appears in the word cloud the same number of times it was identified in the data to provide a visual representation of the frequencies.

**Table 2:** Qualitative analysis of survey responses TdP = Torsades de Pointes; ECG = electrocardiogram; CDS = clinical decision support; BPA = best practice advisory; PCP = primary care provider

Theme	# of times it appears in data	Saturation (out of 21 codes)	Quote
Driven by pharmacist	7	0.33	“The psychiatric clinical pharmacist assesses QTc, current medications, and other risk factors for TdP and makes recommendations to the treatment team based on a patient's risk. The recommendations of the psychiatric pharmacist are almost always implemented."
Driven by non-pharmacist	3	0.14	“Usually done by the prescriber if they feel it is relevant. Pharmacists can only call and suggest to the provider if they feel the need for an ECG.”
Driven by CDS	3	0.14	“A few years ago a pharmacy resident at our parent acute care hospital took our protocol, again gathered data from other facilities and updated our overall policy and developed a QTc monitoring protocol which was then built into our EPIC system which alerts our physicians and pharmacists when patients are at potential risk for QTc prolongation."
Formal process	4	0.19	“QTc Algorithm - A stepped-based algorithm was developed and tested for content validity, with positive results. This is now going to be implemented into a decision support tool.”
Medication-specific	2	0.10	“The only med-specific protocol is for citalopram (that pharmacy can employ). Other than that, we utilize software programs such as vigilanz and also Epic prebuilds/BPA/pop-up messages upon order verification (ex stating something like methadone and Aricept can both increase QTc consider contacting prescriber to order ECG).”
Baseline ECG	5	0.24	“Patients 50yo and older get a baseline ECG on admission.”
Annual ECG	2	0.10	“Baseline and then annually unless starting clozapine or ziprasidone.”
Risk assessment	7	0.33	“Pharmacist recommends ECG (when not obtained on admission) if patient is on multiple QT-prolonging medications.”
Education	1	0.05	“In the past we have provided a lot of education regarding QT prolongation to our pharmacy team. I still provide ongoing education to our pharmacy department and physicians in regards to QT prolongation monitoring.”
Referral	3	0.14	“Mostly we need to refer them to their PCP to get this completed.”
Barrier	3	0.14	“Getting them done can be tricky as it's not like a lab we can schedule or send patient to. We have to get a nurse from their primary care team to do it and they are very busy with other responsibilities. Seeing patients by telemed so they aren't in the building even also makes it harder.”

## Discussion

This study provides insight into the practice of QTc interval monitoring in patients prescribed psychiatric medications across the United States. The majority of respondents reported that their institution did not have either a formal or informal protocol for QTc interval monitoring. Out of those who reported having a protocol at their institution, approximately half reported that it was a drug-specific protocol. Some medications with high risk for QTc prolongation include citalopram, venlafaxine, amitriptyline, thioridazine, haloperidol, ziprasidone, and clozapine. The APA provides recommendations for monitoring these specific medications, making them strong candidates for drug-specific protocols. While utilizing medication-specific protocols can be helpful, patient-specific risk factors must also be considered when determining the necessity and frequency of QTc interval monitoring. While drug-specific protocols may help to narrow down the number of patients to consider, it is often necessary to look further than the medications. 

The majority of respondents reported that there was a psychiatric clinical pharmacy specialist at their institution; however, most also reported that pharmacists were unable to order ECGs. These findings were also observed in the data from the qualitative analysis. The themes that emerged included pharmacist-driven QTc monitoring, having to refer patients to a different provider for monitoring, and encountering significant barriers to monitoring. Implementation of pharmacist-driven monitoring of the QTc interval has demonstrated the potential to improve patient safety [2,21]. One study showed that pharmacist involvement may increase the appropriateness of monitoring [2]. Another suggested that pharmacists may have an important impact in minimizing risks through their knowledge of QTc prolonging drugs, assessment of risk, awareness of drug interactions that are likely to cause QTc prolongation, and attention to dose reductions in renally eliminated QTc prolonging drugs taken by patients with reduced kidney function [21].

While some pharmacists may express concern about ordering ECGs as they are not typically trained in interpretation, authorization to order ECGs may be an opportunity to improve care for patients prescribed psychiatric medications. This barrier for some pharmacists may be an opportunity for education as ECG readouts typically display the measured QTc value. This barrier could also be an area of future research to determine the pervasiveness and impacts of these concerns. Increasing the scope pharmacists' scope has been shown to reduce QTc interval prolongation and increase appropriate ECG utilization [2,22,23]. Another barrier that should be considered is that pharmacists typically work with a supervising prescriber who must approve their therapeutic interventions. The lack of a consensus best practice around QTc monitoring limits the evidence available and may reduce the number of recommendations that pharmacists feel comfortable making. Based on the findings from this study, the majority of institutions surveyed have psychiatric pharmacy experts available. This result may be skewed as the survey was limited to include only subscribers to the psychiatric pharmacy organization’s email list.

Guidance on QTc monitoring that is currently available from major associations does not include algorithms to help a provider determine the course of action on a patient-specific level [15,16]. Based on the findings from this study, the majority of the respondents reported that their institutions have not adopted either of the guidance frameworks provided by the AHA or APA. While these guidelines do identify patient-specific risk factors such as medical conditions, they do not lay out a pathway for decision-making that would be included in an algorithm. This gap in the existing knowledge base may be one reason for the lack of adoption in the majority of healthcare institutions, as these general guidelines only provide prescribers a place to start when making decisions surrounding QTc monitoring. Another limitation of the AHA guidelines is that the majority of Class I recommendations are based on Level of Evidence C. 

While not recognized by major associations, there is published guidance available that provides a more step-by-step approach to QTc monitoring [24-26]. One is a literature review conducted by pharmacists in Qatar and Canada which included 31 articles to create an algorithm for the assessment, management, and monitoring of drug-induced QTc prolongation in psychiatric patients [24]. While this algorithm has been validated, it has not been recognized by major associations [27,28]. The second guidance cited is from the Association of Medicine and Psychiatry and also includes an algorithm determined by a literature search and review of AHA, APA, and other society guidelines [25]. This algorithm is likely not recognized by major associations as it was published only one year prior to the APA guideline update. The third guidance document cited above is from a group of researchers in the United Kingdom and includes an algorithm along with a list of patient risk factors, medications with a risk of QTc prolongation, and a list of QTc prolongation assessment tools [26]. Similarly to the previous algorithm discussed, it is also likely not recognized by major associations as it was published only one year prior to the APA guidelines update. The recent publication of the last two protocols does suggest that research is being conducted on this issue and there may be progress in the near future. However, the current absence of consensus around these monitoring options contributes to the lack of consistent methodology and procedure across healthcare institutions and further highlights the need for a best practice.

Some limitations of this study include low and variable response rates and lack of generalizability due to the population to which the survey was distributed. The small sample size and use of a national psychiatric organization’s email list may have introduced selection bias and limited the generalizability of the results. Future studies may achieve a more representative sample by distributing a survey to pharmacists not involved in the organization. The survey was distributed in a way to reach as many psychiatric pharmacists as possible in one step and included all responses. Due to this, there was no perspective from other providers or pharmacy professionals from outside of the psychiatric specialty. Another limitation is that it is possible that multiple participants from the same institution may have provided differing opinions or that participants may have misrepresented their institutions. Future studies may consider including information on the survey about the institutions such as the types of patients they serve, their location, size, and other demographic information to provide more insight into how these factors may have influenced responses. 

## Conclusions

Further research is needed to more clearly decipher the best practices around QTc prolongation risk mitigation in patients receiving psychiatric medications. This research could be focused on collecting more information on methods currently in use, comparing the efficacy of current methods, or creating new methods to be validated and tested in practice. Based on the findings of this study, there is a need for stronger guidance that can be protocolized by healthcare institutions in the United States in order to decrease variability in QTc interval monitoring for patients prescribed psychiatric medications.
